# Supervised learning of gene-regulatory networks based on graph distance profiles of transcriptomics data

**DOI:** 10.1038/s41540-020-0140-1

**Published:** 2020-06-30

**Authors:** Zahra Razaghi-Moghadam, Zoran Nikoloski

**Affiliations:** 1 Bioinformatics, Institute of Biochemistry and Biology, University of Potsdam, Karl-Liebknecht-Str. 24-25, 14476 Potsdam, Germany; 2 Systems Biology and Mathematical Modeling group, Max Planck Institute of Molecular Plant Physiology, Am Mühlenberg 1, 14476 Potsdam, Germany

**Keywords:** Systems biology, Computational biology and bioinformatics

## Abstract

Characterisation of gene-regulatory network (GRN) interactions provides a stepping stone to understanding how genes affect cellular phenotypes. Yet, despite advances in profiling technologies, GRN reconstruction from gene expression data remains a pressing problem in systems biology. Here, we devise a supervised learning approach, GRADIS, which utilises support vector machine to reconstruct GRNs based on distance profiles obtained from a graph representation of transcriptomics data. By employing the data from *Escherichia coli* and *Saccharomyces cerevisiae* as well as synthetic networks from the DREAM4 and five network inference challenges, we demonstrate that our GRADIS approach outperforms the state-of-the-art supervised and unsupervided approaches. This holds when predictions about target genes for individual transcription factors as well as for the entire network are considered. We employ experimentally verified GRNs from *E. coli* and *S. cerevisiae* to validate the predictions and obtain further insights in the performance of the proposed approach. Our GRADIS approach offers the possibility for usage of other network-based representations of large-scale data, and can be readily extended to help the characterisation of other cellular networks, including protein–protein and protein–metabolite interactions.

## Introduction

Characterisation of gene-regulatory networks (GRNs) remains one of the key challenges in systems biology^1,2^. Successful solution strategies to uncover the determinants of gene expression can be used to understand how genes regulate downstream processes (e.g., signalling, metabolism) and complex phenotypes (e.g., growth, survival, disease susceptibility)^3^. At the simplest level, regulation of gene expression is characterised by binding of a transcription factor (TF) to a promoter region of the target gene and its concomitant activation or repression. Variation in responsiveness of a target gene to a TF, due to genetic variation, change in the environment or a combination thereof, can affect its expression and the resulting cellular phenotype. That said, gene expression is regulated by additional factor that affect gene expression (e.g., degradation)^4^. Bioinformatics studies have used gene expression data collected under steady-state conditions^3^, different time domains in response to external perturbation^5^ and their combination^6^, to reconstruct GRNs (i.e., TF-target gene-regulatory relationships) with different degrees of success. Combining the results from these approaches has been found to perform well for unicellular organisms, such as *Escherichia coli* and *Saccharomyces cerevisiae*^6^. These computational approaches can be validated by and facilitate the integration of experimental data on (putative) binding of a TF to a promoter region of a target gene based on different technologies^7–9^. For instance, Chromatin immunoprecipitation combined with sequencing (ChIP-Seq) facilitates determining functional binding of a TF to a promoter, yeast one hybrid (Y1H) can be employed to identify the proteins that bind a given DNA sequence in vivo^10^, and DNA-affinity purification sequencing (DAP-Seq) helps specify genome-wide TF-binding sites^11^. Although useful and very informative, the data from these technologies are either condition-specific (e.g., ChIP-Seq and Y1H) or may not indicate functional binding of a TF to a gene promoter (e.g., DAP-Seq), leading to a sizeable fraction of false positives when identifying gene-regulatory interactions. In addition, these methods are still resource-intensive even for well-studied model organisms. As a result, it is necessary to further develop approaches that can be used to accurately reconstruct GRNs from expression data^12^.

The computational approaches for GRN reconstruction can be broadly divided into two types: unsupervised, which only rely on availability of gene expression data, and supervised, which in addition to transcriptomics profiles also use knowledge on known gene-regulatory interactions. The supervised approaches are based on inductive reasoning to predict new interactions, whereby if one TF is known to regulate a gene, then all TF-gene pairs with similar features are likely to interact as well. To this end, the expression data profiles for a TF–gene pair are transformed into feature vectors and provided as input to a supervised learning method. The learning method is used to train a classifier which is in turn employed to identify whether or not a pair of genes is involved in a regulatory interaction. A major challenge in supervised learning of GRNs is that there is often no experimental evidence for lack of interaction between a TF and a gene. The latter makes it difficult to use well-established computational approaches aimed for building binary classifiers.

A comprehensive comparative study with synthetic and experimentally obtained transcriptomics data sets has indicated the superiority of supervised over unsupervised approaches for GRN reconstruction^2^. The existing supervised approach for GRN reconstruction, called SIRENE, is based on support vector machines (SVMs)^13^. SIRENE learns a binary classifier that for each TF distinguishes target from non-target genes^14^. A similar SVM-based approach was proposed and employed by Cerulo et al.^15^. SIRENE overcomes the absence of non-interacting pairs of TF and target genes in the following way: For any given TF, it takes the set of genes with which the TF has no reported regulatory interactions, and splits this set into three subsets of roughly equal size. It first sets aside one subset, and then trains an SVM classifier using all known positive instances and the two other subsets, treated as negative instances. Then, this SVM is used to score the instances in the subset left apart. This scoring procedure is repeated, each time setting apart one of the other two subsets, and the scores of the instances are aggregated. SIRENE was tested on an *Escherichia coli* benchmark data set and was shown to outperform the state-of-the-art unsupervised inference methods, including context likelihood to relatedness (CLR)^16^, algorithms for the reconstruction of accurate cellular networks (ARACNE)^17^, relevance networks^18^ and Bayesian networks^19^.

Supervised learning approaches for GRN reconstruction can be further grouped into local and global^20^. In local approaches, a classifier is built to discriminate the target of each TF separately. In contrast, global approaches use all TF-target gene pairs to learn a classifier for gene-regulatory interactions. The global approaches are more suitable for practical applications, since the learned classifier can be used on any TF–gene pair. In such a way, one avoids the possibility of not having sufficient prior knowledge on interactions for a given TF to learn a good classifier (e.g., due to lack of information about its targets). Therefore, design of accurate global approach for GRN reconstruction will allow identification of TFs act as master regulators of multiple processes, and those TFs have fine-tuning role and are involved in regulation of few selected processes.

A class of recent approaches for unsupervised GRN reconstruction are based on random forests (RFs)^21,22^. GENIE3 is an unsupervised approach which decomposes the GRN inference problem into several regression problems and uses tree-based ensemble methods to predict the expression pattern of a target gene from the expression patterns of all remaining genes (e.g., coding for TF)^22^. iRafNet builds on GENIE3 by integrating information provided by other biological data such as protein–protein interactions and expression from perturbation experiments (e.g., knockout studies)^21^. While increasing the predictive power of GENIE3, iRafNet requires consideration of external knowledge from special experimental set-up. These approaches have been widely used due to their good performance in GRN reconstruction on synthetic data sets.

Here, we propose a global SVM-based supervised approach, termed GRADIS, to infer GRNs from genome-wide expression data and known regulatory interactions. Unlike the existing supervised approach, the feature vectors in GRADIS are provided by graph distance profiles from a network representation of the gene expression data. To evaluate the performance of GRADIS, we apply it to synthetic data as well as two benchmark data sets of *Escherichia coli* and *Saccharomyces cerevisiae* provided by the Dialogue for Reverse Engineering Assessments and Methods (DREAM4 and DREAM5) network inference challenges^6,23^. The results demonstrate that GRNs inferred by GRADIS are of higher accuracy, assessed by the area under the ROC curve and the area under the precision-recall curve, in comparison with all other existing supervised learning approaches for GRN reconstruction, the most widely used unsupervised approaches (i.e., CLR, ARACNE, GENIE3, iRafNet, mrnet^24^ and TIGRESS^25^), and their combination following ensemble learning strategies.

## Results

### Formulation of the GRADIS approach

**Fig. 1:**
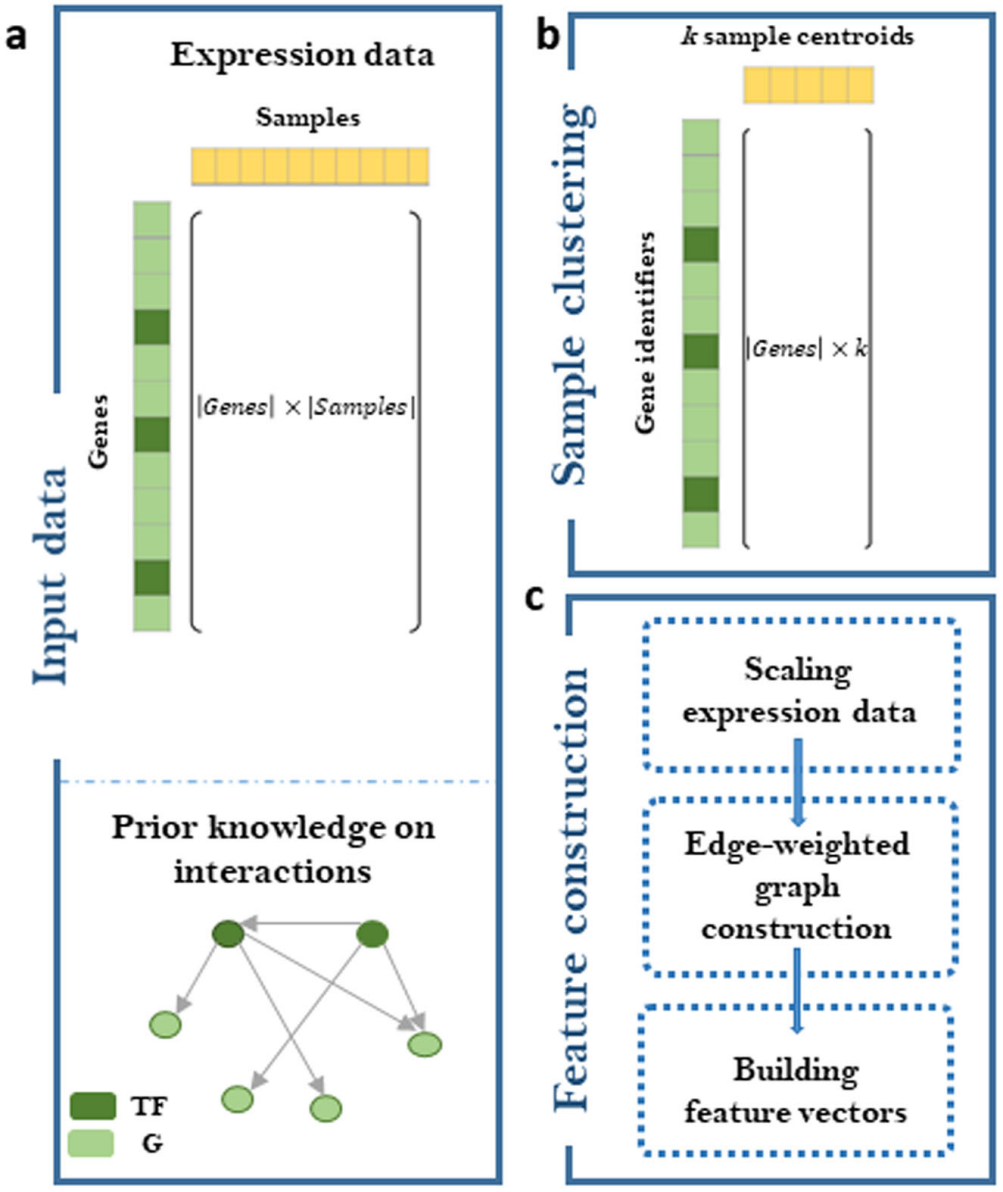
Visualisation of the steps in GRADIS. **a** GRADIS requires expression data and knowledge of known transcription factor (TF) and gene (G) interactions as input. **b** The samples in the expression data are first clustered using k-means clustering, and the respective centroids are used to obtain informative and non-redundant data. **c** Features are then constructed from the scaled data sets obtained from the sample clustering in (**b**).

Our GRADIS supervised approach is based on graph distance profile to infer regulatory interactions between TFs and all (TF and non-TF-coding) genes in an organism of interest. GRADIS consists of three main steps: (1) sample clustering, whereby samples with similar expression profiles are first partitioned into *k* clusters (e.g., based on *k*-means clustering algorithm^26^); (2) Euclidian-metric graph construction, whereby the expression profiles obtained from step (1) for each TF–gene pair are cast as a Euclidean-metric complete graph, where the gene can either encode TF or non-TF; (3) SVM-based classification, whereby a binary classifier for the TF–gene pair is trained based on the graph distance profile from step; (2) to discriminate target from non-target genes (for a visual illustration of GRADIS, see Fig. 1). A key step of GRADIS is the construction of the Euclidian-metric complete graph, which provides the key difference to supervised approaches for GRN reconstruction. Since the pair TF–gene is an ordered pair between a regulator and a regulated gene, the inferred statistical relationships from GRADIS can be considered as directed.

To train a binary classifier, the input data set should contain positive and negative instances. However, available experimentally verified networks provide little information regarding the absence of regulations between a TF and a gene. Another distinguishing aspect of the GRADIS is the technique used to generate negative instances during the SVM classification subtask. In what follows, we provide the details of the three steps.

### Step 1: sample clustering

The number of samples used in GRADIS determines the number of features used in the SVM-based classification. To provide informative, non-redundant features, a pre-processing step is needed to cluster the data samples into a smaller number of clusters based on their similarity. This step differs from determination of clusters based on genes, applied in other GRN reconstruction approaches^27^. To this end, GRADIS employs the *k*-means clustering algorithm, so that the original data samples are grouped into *k* clusters. We use *k*-means clustering, since it allows us to investigate the effect of the cluster numbers, for any number of clusters *k*, on the performance of GRADIS. The resulting cluster centroids are then gathered in a new data set that effectively summarises the information in the original one. In this reduced data set, the expression profile of gene *g* is a *k*-dimensional vector ${\mathbf{x}}_g = ( {x_g^1,x_g^2, \ldots ,x_g^k})$, where $x_g^i\left( {i = 1,2, \ldots ,k} \right)$ is the expression level of gene *g* in the cluster centroid *i*.

### Step 2: construction of the graph distance profile

To provide a global supervised approach for GRN reconstruction, we next build a feature vector for a TF–gene pair based on the respective expression profiles. The expression profiles are obtained from the sample clustering step, above. To account for the magnitude differences among transcript levels of the TF gene and the putative target, the expression profiles are rescaled to lie in the interval [0, 1].

**Fig. 2:**
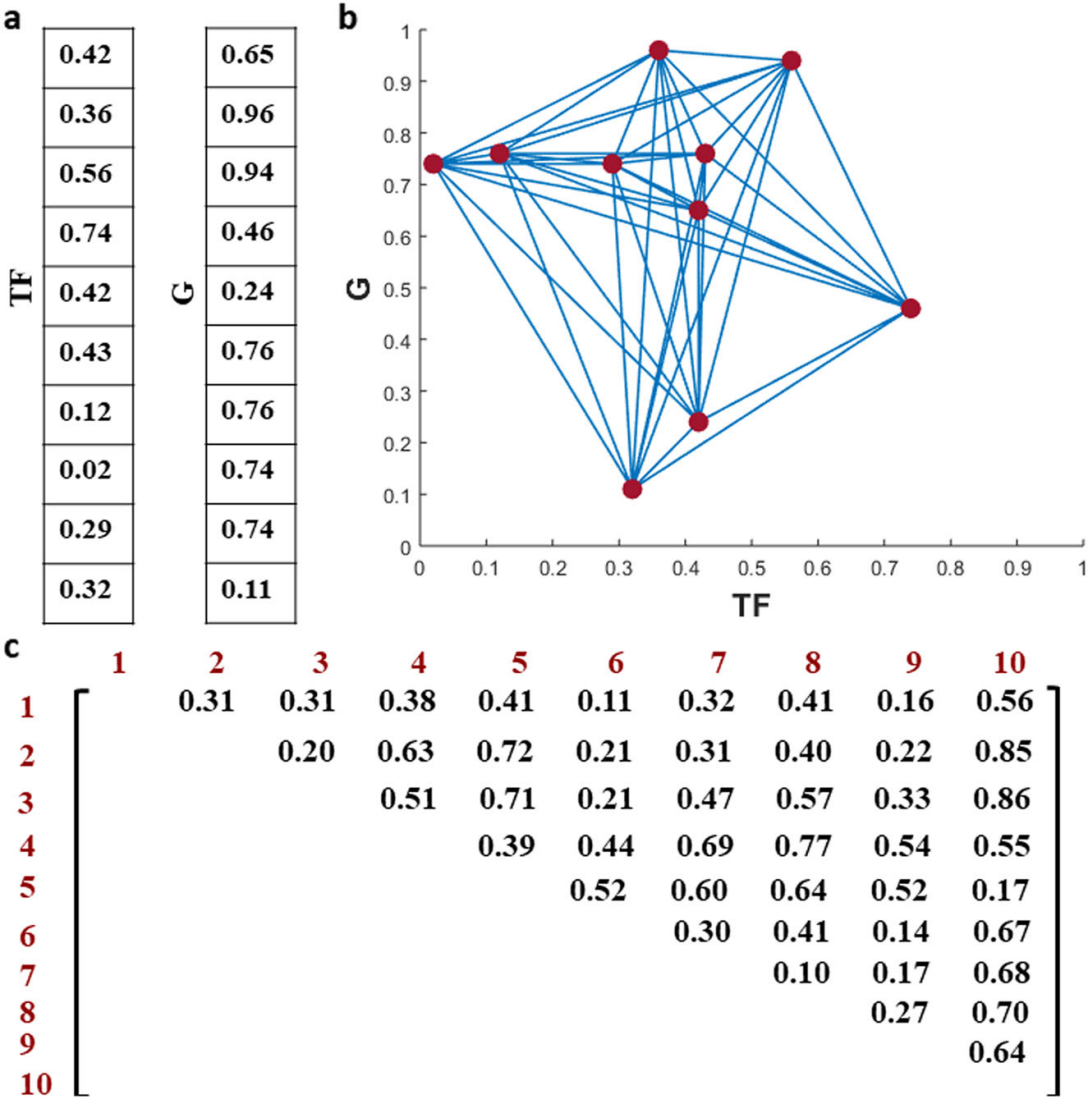
Construction of a Euclidian-metric complete graph. An example of expression profiles (**a**) of transcription factor (TF) and a gene (*G*) represented in the unit square (**b**), and (**c**) the adjacency matrix of the Euclidean-metric complete graph obtained from (**b**). The feature for the TF–gene pairs is given by vectorisation of the upper triangle of the matrix (excluding the diagonal as non-informative).

A pair of scaled *k*-dimensional vectors can be depicted by *k* points in the unit square. Using a mathematical notation, the gene pair (TF, *G*) with expression profiles of ${\mathbf{x}}_{\mathrm{{TF}}} = (x_{{\mathrm{TF}}}^1,x_{{\mathrm{TF}}}^2, \ldots ,x_{{\mathrm{TF}}}^k)$ and ${\mathbf{x}}_G = (x_G^1,x_G^2, \ldots ,x_G^k)$ can be represented by the *k* points $\left( {x_{{\mathrm{TF}}}^1,x_G^1} \right),\left( {x_{{\mathrm{TF}}}^2,x_G^2} \right), \ldots ,\left( {x_{{\mathrm{TF}}}^k,x_G^k} \right)$ in the unit square (Fig. 2a, b).

In the next step, we create a complete edge-weighted graph for each pair using its corresponding set of *k* points as nodes (Fig. 2b). The weight of the edge connecting nodes $n_i = \left( {x_{{\mathrm{TF}}}^i,x_G^i} \right)$ and $n_j = ( {x_{{\mathrm{TF}}}^j,x_G^j})$ (*i* ≠ *j* and 1 ≤ *i*, *j* ≤ *k*) is defined as the Euclidian distance between the two points, given by $w( {n_i,n_j}) = \sqrt {(x_{{\mathrm{TF}}}^i - x_{{\mathrm{TF}}}^j)^2 + (x_G^i - x_G^j)^2}$. Having formed this weighted graph, the upper right triangle of the weighted adjacency matrix, excluding the diagonals, is then utilised as a feature vector. As the weighted adjacency matrix is of size *k* × *k*, the upper right triangle has *k*−*i* (1 ≤ *i* ≤ *k*) entries in its *i*th row. Hence, concatenating all the rows of the triangle into one feature vector leads to an array of length$$\left( {\begin{array}{*{20}{c}} k \\ 2 \end{array}} \right) = \mathop {\sum}\nolimits_{i = 1}^k {\left( {k - i} \right)}$$, which is subsequently used to learn a binary classifier (Fig. 2c). This feature vector captures the statistical relationship between samples, which is not considered in approaches that rely on similarity measures to determine statistical relationships between the levels of a TF and putative target gene. Therefore, this unique representation of between-sample relationships provide additional information in accurately reconstructing GRNs. The feature vectors are formed based on the Euclidean distance, since it is a widely used natural distance metric. However, for the purpose of comparison, we also apply Manhattan distance to compute the edge weights and further construct the features. The Manhattan distance for a pair of nodes $n_i = \left( {x_{{\mathrm{TF}}}^i,x_G^i} \right)$ and $n_j = ( {x_{{\mathrm{TF}}}^j,x_G^j})$ (*i* ≠ *j* and 1 ≤ *i*, *j* ≤ *k*) is given by $w_m( {n_i,n_j}) =| {(x_{{\mathrm{TF}}}^i - x_{{\mathrm{TF}}}^j)}| +| {(x_G^i - x_G^j)}|$.

### Step 3: SVM classification

SVM is a well-known binary classifier of points that belong to either the positive or the negative class^28^. In GRADIS, the positive class of points is given by pairs of TF and a confirmed target, while the negative class is provided by TF-non-target gene pairs. Training of the SVM then utilises feature vectors obtained from the Euclidian-metric graph for the pair of TF and gene. In addition, we employ the feature vectors obtained from the Manhattan distance to train an SVM and to compare the results between the two distance metrics.

Typically, there is little information available about the absence of gene-regulatory interactions between TFs and target genes in real-world data sets. Hence, it is not straightforward to train a classifier for this specific problem due to lack of negative instances. To overcome this issue, GRADIS utilises the following labelling strategy to identify potential members of the negative class: Naturally, the prior experimentally characterised TF–gene interactions (e.g., by ChIP-seq and other technologies), included in a gold-standard network, comprise the positive class of the training data. The uncharacterised TF–gene pairs then are divided into several subsets of size equal to that of the positive class. At each iteration, only one of these subsets is treated as the negative class, which is used together with the positive class to train a new, iteration-specific SVM using tenfold cross-validation on the positive and negative class selected in the iteration. All the uncharacterised pairs in the remaining subsets are in turn treated as test data in this iteration, which are to be assessed by this specific SVM classifier. These partial assessments by the individual classifiers trained in each iteration are finally aggregated to make a final decision on our choice of potential negative instances.

The adoption of this labelling strategy leads to training as many as $\frac{{\left| {{\mathrm{uncharacterized}}\,{\mathrm{pairs}}} \right|}}{{\left| {{\mathrm{positive}}\,{\mathrm{pairs}}} \right|}}$ independent SVM classifiers. Initially, a zero score is assigned to all uncharacterised TF–gene pairs. The score for an uncharacterised TF–gene pair is subsequently updated in each iteration as follows: the trained iteration-specific SVM classifier categorises each uncharacterised TF–gene pair in the test set to be either positive or negative. If the prediction for a pair is positive, its score is incremented by one; otherwise, the score remains unchanged. This procedure is then repeated in the next iteration by taking another subset of the uncharacterised TF–gene pairs as the negative set, and classifying the remaining such pairs. Eventually, each uncharacterised TF–gene pair will end up with a certain score obtained through this process, which reflects the plausibility of an interaction existing between these genes. Intuitively, a lower final score for a TF–gene pair indicates a higher likelihood that it belongs to the negative class. The negative class for the training data is then constructed by selecting those pairs with a zero final score. The number of the negative instances found with this approach is considerably higher than the number of positive ones.

Having obtained a labelled training set associated with the feature vectors explained earlier, an SVM can be trained to find an optimal hyperplane that separate the two classes. The training set consists of *n* TF–gene pairs *p*_1_, *p*_2_,…, *p*_*n*_, each of which belong to either of the two positive and negative classes, respectively denoted by +1 and −1. Once the SVM classifier is trained, it can predict the label (class) of any uncharacterised TF–gene pair *p*. This labelling is done by SVM based on a scoring function of the form$f\left( p \right) = \mathop {\sum}\nolimits_{i = 1}^n {\alpha _iK\left( {p_i,p} \right)}$. The *α*_*i*_ are Lagrange multipliers, which are optimised by SVM to enforce large positive scores for gene pairs in the +1 class and large negative scores for pairs in the −1 class in the training set. The kernel function *K*(·,·) is a basic component of the SVM, which provides an implicit mapping of data points into a high-dimensional space, in which the optimal hyperplane can be obtained. In GRADIS, the SVM classifier is trained with a Gaussian (RBF) kernel function. GRADIS is implemented in Matlab R2017b and is available online at https://github.com/MonaRazaghi/GRADIS. To examine the extent to which the choice of machine-learning algorithm affects the performance of the GRN reconstruction, we also train RFs^29^ on feature vectors obtained from the Euclidian-metric graph and compare its results with those of the SVM.

### Comparative analysis

To assess the performance of the proposed approach, GRADIS, and compare it with the contending approaches, we used the area under the ROC curve (AUC) and the area under the precision-recall curve (AUPR^30^) obtained from synthetic and real-world data sets for which gold-standard interactions are available from the DREAM4 and DREAM5 challenges. To ensure robustness of our findings and obtain highly reliable AUC and AUPR measures, a tenfold cross-validation with ten repetitions is performed for the supervised approaches (see Supplementary Table S4 for the sizes of training and testing data sets). As indicated above, the positive and negative classes are not balanced, in the sense that there are considerably more negative than positive instances. Such a lack of balance in the size of classes may lead to training a classifier that is biased towards the bigger class. To avoid this issue in GRADIS, we ensure that each fold on which the SVM classifier is trained contains the same number of instances from both classes. This is achieved by considering all positive instances in the selected training set (from the tenfold cross-validation) and sampling the same number of negative instances uniformly at random.

### Effects of the number of clusters

An initial step of GRADIS implementation involves selecting the number of clusters, *k*, to use only the most informative samples. To gauge the selection of an appropriate value for *k*, we first investigate the effect of this parameter on the performance of GRADIS. We observe that higher values of AUC are generally associated with larger values of *k* for all three data sets from the DREAM5 challenge (Supplementary Fig. S1). In addition, as we increase the number of clusters, *k*, the value of AUC approached its maximum at about *k* = 50. However, further increase in the value of *k*, above 50, does not result in a noticeable improvement in the value of the AUC (Supplementary Fig. S1).

For fairness of comparison, we also make sure that the number of clusters used in GRADIS ensures the usage of a similar number of features as the approaches based only on the expression profiles. Specifically, for the synthetic data from the DREAM4 challenge, the 210 samples are clustered in *k* = 30 clusters, yielding 435 features for GRADIS. This is similar to the 420 (=2.210) features used by the other approaches. In addition, for the synthetic data from the DREAM5 challenge, the 805 samples are clustered in *k* = 50 clusters, yielding 1225 features for GRADIS.

### Comparison of performance with synthetic data

We first compare the performance of GRADIS with that of the other contenders on synthetic data sets for which both positive and negative interactions instances are known. To this end, we use six popularly used unsupervised approaches, CLR, ARACNE, GENIE3, iRafNet, mrnet and TIGRESS, and supervised approach SIRENE and an expression-based SVM classifier. Because of the local strategy exploited in SIRENE, for this approach we only train local classifiers for each TF, together with a corresponding value for AUC. Finally, we use the average AUCs in the comparisons. In addition, we consider combining the information of the unsupervised approaches following the wisdom of crowds strategy^6^.

As shown in Table 1, GRADIS outperforms all contending approaches based on the average AUC, over the different classifiers with balanced number of positive and negative instances, when using the synthetic data from the DREAM4 and DREAM5 challenges. Interestingly, for all synthetic data, except for Network 4 from the DREAM4 challenge, GRADIS also performs better than the wisdom of crowds that combines the findings of CLR, ARACNE, GENIE3, iRafNet, mrnet and TIGRESS. Therefore, we conclude that the features used in GRADIS provide a considerable advantage to the other computational approaches. Since iRafNet is based on RF, we are also interested to investigate if the better performance of GRADIS is due to the differences between the methods used to train the classifiers (i.e., SVM vs. RF). To this end, we compare the performance of GRADIS with that of an expression-based SVM classifier that employs the expression of TFs and target genes. For all synthetic networks, the average AUC of GRADIS is larger than that of the expression-based SVM classifier. Further, we observe that: (1) the upper bound of the confidence intervals for the AUC of GRADIS is consistently larger than that of the expression-based SVM classifier, and (2) the lower bound of the confidence intervals for the AUC of GRADIS is at least as large as the average AUC of the expression-based SVM classifier over the different networks. Altogether, these findings demonstrate the robustness of the excellent performance of GRADIS.

**Table 1:** Comparative analysis based on area under the ROC curve (AUC).

Data	Methods									
	ARACNE	CLR	TIGRESS	mrnet	GENIE3	iRafNet	Wisdom of crowds	SIRENE (average)	Expression-based SVM	GRADIS
DREAM4										
* Net1*	0.56	0.71	0.50	0.69	0.77	0.5	0.82	0.54	0.81 (0.77–0.84)	0.86 (0.80–0.92)
* Net2*	0.54	0.64	0.50	0.65	0.69	0.5	0.78	0.48	0.83 (0.79–0.87)	0.85 (0.82–0.88)
* Net3*	0.56	0.71	0.52	0.72	0.73	0.5	0.79	0.5	0.72 (0.66–0.77)	0.77 (0.72–0.82)
* Net4*	0.55	0.67	0.51	0.67	0.69	0.5	0.78	0.5	0.70 (0.67–0.73)	0.76 (0.72–0.80)
* Net5*	0.58	0.68	0.51	0.52	0.76	0.5	0.80	0.48	0.71 (0.64–0.79)	0.77 (0.71–0.82)
DREAM5										
* InSilico*	0.50	0.50	0.74	0.74	0.82	–	0.81	0.42	0.84 (0.83–0.85)	0.85 (0.84–0.86)
* E. coli*	0.51	0.59	0.59	0.59	0.69	–	0.69	0.41	0.87 (0.85–0.88)	0.94 (0.93–0.94)
* S. cerevisiae*	0.50	0.52	0.52	0.52	0.54	–	0.54	0.49	0.80 (0.79–0.81)	0.96 (0.96–0.97)

The performance of GRADIS is compared with that of unsupervised approaches (ARACNE, CLR, GENIE3, iRafNet, mrnet, TIGRESS), their combination based on wisdom of crowds and two supervised approaches (SIRENE and expression-based SVM classifier). Since the performance is based on the global (i.e., network-centric) approach, for SIRENE we report the average AUC over all TFs (for local comparison, refer to ‘Methods'). The numbers in parentheses refer to confidence intervals (see ‘Methods'). The comparison includes the five synthetic data sets from the DREAM4 challenge as well as the one synthetic and the two real-world data sets from the DREAM5 challenge. Results from iRafNet are not provided for the data sets in DREAM5 due to lack of data on knockout experiments and protein–protein interactions.

Similar findings hold for the comparison of the approaches based on the AUPR statistic. GRADIS outperforms all other contending approaches and their combination based on the wisdom of crowds (Supplementary Table S1). For all networks, except Network 2 from the DREAM4 challenge, GRADIS outperforms the expression-based SVM classifier.

Furthermore, we apply the Manhattan distance to compute the edge weights in the graph representation of our data and compare the findings with those from the Euclidean distance. The results show the coefficients of correlation between Manhattan distance and Euclidean distance vary from 0.96 to 0.99, based on Mantel test^31^. The high correlation between the two distance metrics implies that the SVM with Manhattan distance performs similarly to that based on the Euclidean distance (Supplementary Table S2). Eventually, to evaluate the impact of classification algorithms, we also train RF classifier based on Euclidean-metric graph and compare its performance to that of SVM classifier. The results show that using the graph-based features, SVM classifier performs better than random forests in reconstructing GRNs (Supplementary Table S3).

### Comparison of performance with real-world data

The findings from the synthetic data sets have motivated us to explore the performance of GRADIS on real-world expression data sets from *E. coli* and *S. cerevisiae* provided in the DREAM5 challenge. Here, we first learn negative interaction instances, and use them to train a global classifier. We find that for the two real-world networks in the DREAM5 challenge, GRADIS outperforms each of the contending approaches, individually as well as their combination based on the wisdom of crowds strategy (Table 1). Moreover, GRADIS outperforms the expression-based SVM with respect the average AUC; further, the confidence intervals do not overlap, strengthening the claims about the better performance of GRADIS. Similar findings hold with respect to the average AUPR (Supplementary Table S1). We note that no results could be obtained based on iRafNet as this approach requires data from knockout experiments or protein–protein interaction data, which are not provided in the DREAM5 challenge.

### Comparison of local and global approaches

Although GRADIS and SIRENE are both based on SVM, they adopt two intrinsically different strategies to GRN inference, namely the global and local, respectively. Hence, to provide a fair assessment, we compare GRADIS and SIRENE following a TF-centric (i.e., local) and network-centric (i.e., global) perspective. Supplementary Fig. S2 presents the network-centred results obtained from SIRENE, applied on each of the two real-world data sets. Both ROC and PR curves in Supplementary Fig. S2 indicate that GRADIS outperforms SIRENE from a network-based (global) perspective. To compare the two approaches from the TF-centred (local) perspective, we use the GRADIS results to calculate the AUC for each individual TF. We note that in this approach, some TFs may not be present in the test set, thereby we repeat the analysis ten times. We then calculate the distribution of AUC values based on the minimum, maximum and mean values from the ten repetitions. Finally, we compare the performance of two approaches via box plots, as shown in Supplementary Fig. S3. The results of this local investigation once again show that GRADIS has a superior performance compared with SIRENE. For instance, we find that the median values for the distributions of minimum, mean and the maximum AUC values over all TFs are consistently larger for GRADIS on the data sets of *E. coli* and *S. cerevisiae*.

### Effects of determining the negative class

In the aforementioned scoring scheme from the SVM classification, a higher score for a pair is indicative of a positive class instance, i.e., of a regulatory interaction. To further evaluate the proposed strategy for determining negative class instances, the uncharacterised pairs that are less likely to be negative instances are selected, based on a given threshold score, and cross-examined with an experimentally verified database. For instance, for the *E. coli* data set, 80 $\left( { = \frac{{\left| {{\mathrm{uncharacterized}}\,{\mathrm{pairs}}} \right|}}{{\left| {{\mathrm{positive}}\,{\mathrm{pairs}}} \right|}}} \right)$ SVM classifiers were trained to identify pairs that may serve as negative instances. Interestingly, the results show that 49 (out of 223,262) uncharacterised pairs received the maximum possible score of 80. The pairs which received a score above 75 (7728 pairs) were selected and cross-examined with RegulonDB. From the 7728 pairs with score larger than 75, we find that 275 represent true gene-regulatory interactions. To assess the significance of this finding, we generated a null distribution of 1000 sets of random uncharacterised pairs of the same size (7728), and examined their interactions in RegulonDB. The maximum number of true regulatory interactions among the 1000 random sets is 63, which is considerably smaller than 275. This further indicates that pairs with high scores are indeed significantly enriched for regulatory interactions (*P* < 0.001).

To evaluate the performance of this scoring scheme for *S. cerevisiae*, we test the same strategy on regulatory interactions with DNA-binding evidence from YEASTRACT. Here, the results show that 32 (out of 56,281) uncharacterised pairs receive the maximum possible score of 56. The maximum number of true regulatory interaction among the 1000 random sets is 29, which further supports the validity of the proposed strategy in providing information about uncharacterised TF–gene pairs, particularly for those with highest scores (of 56).

## Discussion

Despite considerable advances in computational approaches to reconstruct cellular networks in model organisms from data profiles of the participating components^32^, there is a considerable gap in our knowledge of GRNs, particularly for eukaryotes. Therefore, further developments of computational approaches is needed to increase the accuracy of the predicted interactions and consequently obtain a larger return on investment in experimental network validation. Our thorough comparative analysis with existing, widely employed unsupervised and supervised approaches for GRN reconstruction from synthetic and real-world gene expression data sets demonstrates that unsupervised approaches perform very close to random guessing of interactions. As a result, here we focus on developing a new supervised approach for GRN reconstruction—a challenge which requires a computational strategy for selecting non-interacting pair of TF and target genes.

The novelty of our supervised approach, termed GRADIS is the usage of network-based representation of the expression data. This network-based representation allows us to capture and later use the relationship of a TF and its target genes between the samples to distinguish interacting from non-interacting TF–gene pairs. In such a way, we obtain a richer representation of the data, rather than a single value, quantifying the relationship between a TF and a gene, obtained by classical similarity measures (e.g., mutual information, different correlation coefficients) with the gene expression profiles. Another novelty of the approach is the way in which we select representative non-interacting TF–gene pairs based on which the final SVM model is trained. Here, we use an iterative scheme to learn putative non-interacting TF–gene pairs. Our comparison with available knowledge of the GRN of *E. coli* and *S. cerevisiae* provides evidence for the validity of the proposed strategy.

Finally, our thorough comparative analysis based on synthetic and real-world data sets demonstrates that the proposed approach GRADIS exhibits considerably better performance than the popularly used unsupervised as well as supervised computational approaches for inference of GRNs. In the comparative analyses, we insisted on fairness with respect to the available data to each of the algorithms. In this sense, we ensured that all algorithms obtained same (or similar) number of features, thus avoiding bias in the comparisons.

The graph-based features can be used as a promising step towards learning other types of molecular interactions, including protein–protein and protein-small molecule (e.g., drug), based on data profiles from other high-throughput profiling technologies. To this end, sparser network representations based on unique geometric graphs on a set of data points can be investigated in future work.

## Methods

### Performance statistics

To assess the performance of GRADIS and compare it with that of other contenders for GRN reconstruction, we use the area under the ROC curve (AUC) and the area under the precision-recall curve (AUPR^30^). To this end, the sensitivity and specificity as well as the precision and recall are calculated for each threshold value, resulting in the ROC and the precision-recall curves, respectively. In addition, we determine confidence intervals for the AUC and AUPR statistics for the SVM-based approaches implemented in this study by first fitting a normal distribution to the statistics obtained from ten different runs. The 95% confidence intervals is then computed based on the resulting distributions.

### Contending computational approaches and wisdom of crowds

We provide a comprehensive comparison of GRADIS to six unsupervised and one supervised approach proposed to date for GRN reconstruction. In addition, we consider another supervised approach based on concatenation of gene expression profiles of TF and target gene, similar to the approach of Ni et al.^33^. For this expression-based SVM classifier, the negative instances are obtained by randomly sampling from the uncharacterised instances. Such an approach allows us to determine the added value of using the graph-based representation of the expression profiles for a TF and a gene, coupled with our strategy for balancing the negative instances. To infer the GRN based on the unsupervised approaches, CLR, ARACNE and mrnet, we employ the implementation in the R package minet. To infer the GRN based on GENIE3, we used the implementation in the R package GENIE3 (https://bioconductor.org/packages/devel/bioc/vignettes/GENIE3/inst/doc/GENIE3.html), and for iRafNet, the R code implementation and a tutorial are obtained from http://research.mssm.edu/tulab/software/irafnet.html. In the case of TIGRESS and SIRENE, we used the MATLAB implementations freely available at http://cbio.ensmp.fr/tigress and http://cbio.ensmp.fr/sirene, respectively. To provide a comparison with another state-of-the-art approach, we also implemented the strategy used by wisdom of crowds (i.e., ensemble learning), whereby integration of the findings from multiple weak classifiers results in more accurate predictions^34^. Wisdom of crowds integrated the findings from six unsupervised approaches (where possible), namely, CLR, ARACNE, GENIE3, iRafNet, mrnet and TIGRESS. Each of these approaches provides a scoring matrix for TFs and genes, comprising the likelihood that a given TF regulates a specified gene. To facilitate integration, each of the scoring matrices was first scaled by dividing with the corresponding maximum score. An interaction was then considered as present, if the associated values in all scaled scoring matrices were above a threshold used for obtaining the AUC and AUPR curves. Results from SERINE are not considered, since the used implementation does not provide the identity of the predicted TF–gene interactions. The results from the other supervised approaches used are not considered in the wisdom of crowds to provide an unbiased comparison between the combination of weak classifiers resulting from the unsupervised approaches.

### Data

We employ five synthetic benchmark data sets from the DREAM4 and one from the DREAM5 challenge to assess GRADIS. These data sets comprise both time-resolved and perturbation experiments. Each of the five synthetic expression data sets from DREAM4 has expression values of 100 genes in 210 time points (i.e., samples), and the number of TF-coding genes in the corresponding gold-standard networks varies from 34 to 44. The synthetic data from DREAM5 comprises the expression values of 1643 genes in 805 samples. The corresponding network has 178 TFs and 4012 regulatory interactions.

Moreover, we use two independent real genome-wide expression data sets and corresponding gold-standard networks to evaluate the performance of GRADIS on real-world data. The expression data with gold-standard networks for *E. coli* and *S. cerevisiae* are obtained from the DREAM5 website. The *E. coli* data set has expression values of 4297 genes in 805 samples. The corresponding network has 2066 regulatory interactions, in which 141 TFs regulate 999 target genes (TF and non-TF-coding genes). The *S. cerevisiae* data set has the expression levels of 5667 genes in 536 samples, and the GRN includes 3940 regulatory interactions between 114 TFs and 1934 target genes (TF- and non-TF-coding genes). The number of available features is one of the factors that affect the performance of the compared approaches.

## Supplementary information

Supplemental Material

## Data Availability

The data sets analysed during the current study are available in the Dialogue for Reverse Engineering Assessments and Methods (DREAM4 and DREAM5) network inference challenges^6,23^.

## Code availability

The implementation is available on GitHub https://github.com/MonaRazaghi/GRADIS.
