# Supplementary material for: Supervised learning of gene-regulatory networks based on graph distance profiles of transcriptomics data
Source: NPJ Syst Biol Appl. 2020 Jun 30;6:21. doi: 10.1038/s41540-020-0140-1 (PMC7327016; doi:10.1038/s41540-020-0140-1)
Supplement: Supplementary file 1 — Supplemental Material [file 41540_2020_140_MOESM1_ESM.pdf]

Supplementary information

Supervised learning of gene regulatory networks based on graph distance profiles of transcriptomics data

Zahra Razaghi-Moghadam[1,2] and Zoran Nikoloski[1,2*]

[1]Bioinformatics, Institute of Biochemistry and Biology, University of Potsdam, Karl-Liebknecht-Str. 24-25, 14476 Potsdam, Germany [2]Systems Biology and Mathematical Modeling group, Max Planck Institute of Molecular Plant Physiology, Am Mühlenberg 1, 14476 Potsdam, Germany

*To whom correspondence should be addressed.

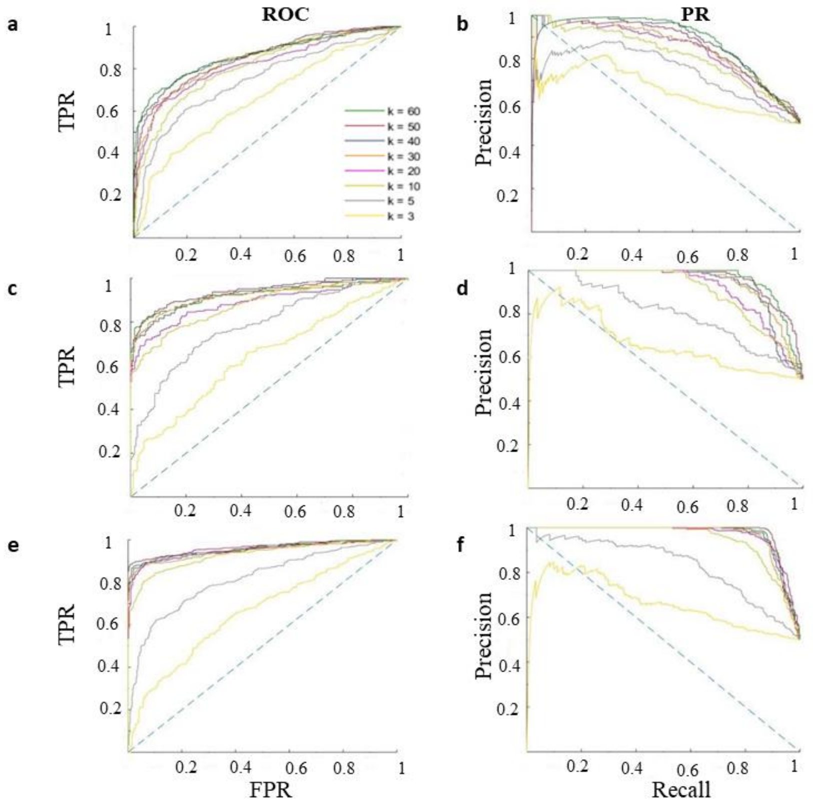

Supplementary Figure 1. The impact of the number of clusters on the performance of GRADIS. (a) ROC curves for different number of clusters (k) for the synthetic data set from DREAM5 challenge, (b) PR (Precision/Recall) curves for different number of clusters (k) for the synthetic data set from DREAM5 challenge, (c) ROC curves for different number of clusters (k) for the E. coli dataset, and (d) PR (Precision/Recall) curves for different number of clusters (k) for the *E. coli* dataset. (e) ROC curves for different number of clusters (k) for the S. cerevisiae dataset, and (f) PR (Precision/Recall) curves for different number of clusters (k) for the *S. cerevisiae* dataset.

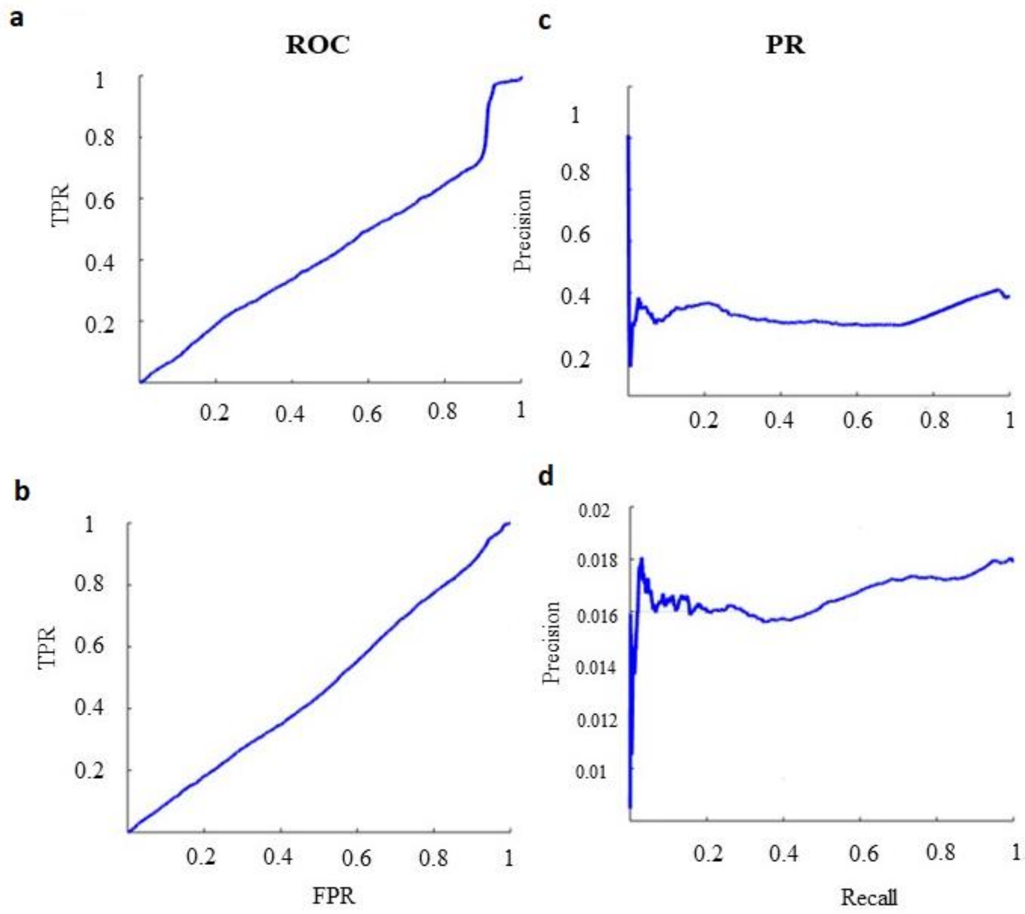

Supplementary Figure 2. Performance of SIRENE from a network-centered (global) perspective. (a) the ROC curve for SIRENE on the *E. coli* dataset, (b) the PR curve for SIRENE on the *E. coli* dataset, (c) the ROC curve for SIRENE on the *S. cerevisiae* dataset, (d) the PR curve for SIRENE on the *S. cerevisiae* dataset. See Figure S1 for comparison to GRADIS.

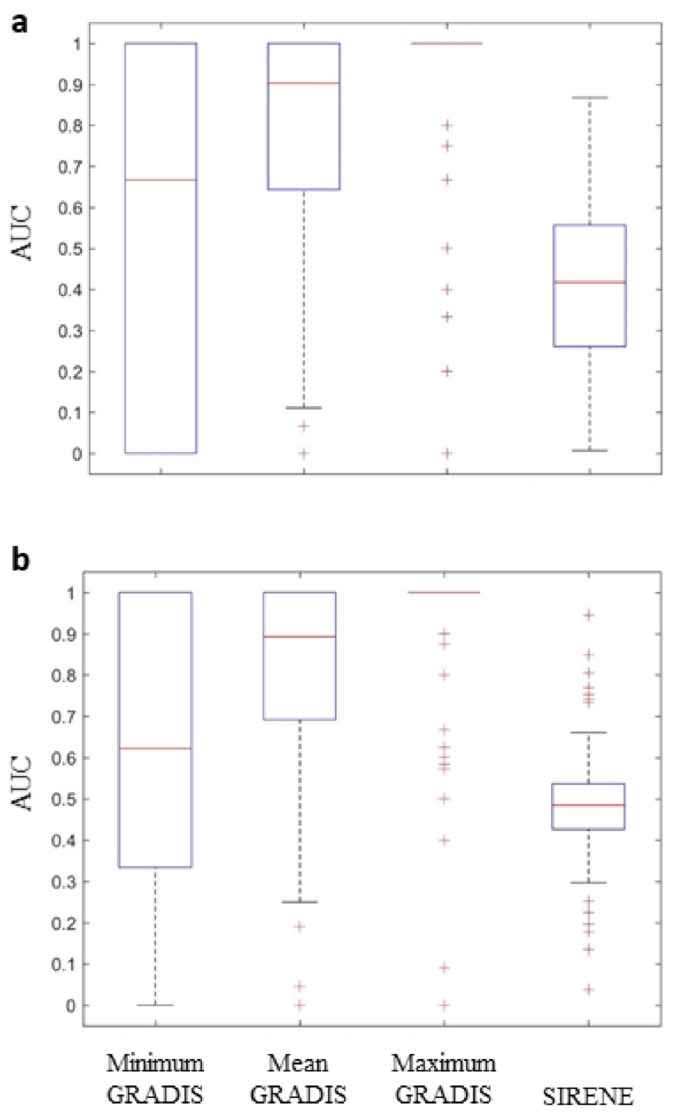

Supplementary Figure 3. Performance of SIRENE based on a TF-centered (local) perspective. The box plots summarize for the distribution of minimum, maximum, and mean AUC values for (a) *E. coli* and (b) *S. cerevisiae***.**

Supplementary Table 1. **Comparative analysis based on area under the Precision-Recall curve (AUPR).** The performance of GRADIS is compared to that of unsupervised approaches (ARACNE, CLR, TIGRESS, mrnet, GENIE3, iRafNet), their combination based on wisdom of crowds, and two supervised approaches (SIRENE and expression-based SVM classifier). Since the performance is based on the global (i.e. network-centric) approach, for SIRENE we report the average AUC over all TFs (for local comparison, refer to Methods). The numbers in parentheses refer to confidence intervals (see Methods). The comparison includes the five synthetic datasets from the DREAM4 challenge as well as the one synthetic and the two real-world datasets from the DREAM5 challenge. Results from iRafNet are not provided for the datasets in DREAM5 due to lack of data on knock-out experiments and protein-protein interactions.

**Data**		**Methods**									
		ARACNE	CLR	TIGRESS	mrnet	GENIE3	iRafNet	Wisdom of crowds	SIRENE (average)	Expression-based SVM	GRADIS
DREAM4	*Net1*	0.09	0.11	0.04	0.11	0.16	0.09	0.06	0.1	0.74 (0.71-0.78)	0.80 (0.74-0.85)
	Net2	0.13	0.13	0.04	0.14	0.14	0.01	0.06	0.11	0.80 (0.76-0.84)	0.78 (0.74-0.83)
	Net3	0.16	0.19	0.06	0.19	0.21	0.01	0.07	0.09	0.64 (0.58-0.70)	0.73 (0.68-0.78)
	Net4	0.12	0.13	0.04	0.13	0.15	0.01	0.06	0.09	0.67 (0.65-0.69)	0.72 (0.67-0.77)
	Net5	0.19	0.18	0.06	0.09	0.21	0.01	0.07	0.1	0.68 (0.61-0.76)	0.74 (0.68-0.80)
DREAM5	*InSilico*	0.01	0.01	0.23	0.21	0.3	-	0.1	0.02	0.85 (0.84-0.87)	0.86 (0.85-0.87)
	E. coli	0.16	0.06	0.01	0.05	0.1	-	0.05	0.02	0.88 (0.87-0.89)	0.94 (0.94-0.95)
	S. cerevisiae	0.02	0.02	0.005	0.01	0.02	-	0.02	0.02	0.79 (0.78-0.80)	0.97 (0.97-0.98)

Supplementary Table 2. **Comparison between GRADIS using Euclidean distance and Manhattan distance based on area under the ROC curve (AUC) and area under the Precision-Recall curve (AUPR).** The extent to which the distance metric affects the performance of GRADIS is assessed by AUC and AUPR. The numbers in parentheses refer to confidence intervals (see Methods). The comparison includes the five synthetic datasets from the DREAM4 challenge as well as the one synthetic data from the DREAM5 challenge.

**Data**		**Methods**			
		GRADIS Euclidean distance		GRADIS Manhattan distance	
		AUC	AUPR	AUC	AUPR
DREAM4	*Net1*	0.86 (0,80-0,91)	0.81 (0.76-0.86)	0.83 (0.76-0.90)	0.79 (0.72-0.86)
	Net2	0.79 (0.87-0.83)	0.78 (0.75-0.82)	0.84 (0.81-0.88)	0.80 (0.76-0.84)
	Net3	0.77 (0.75-0.80)	0.72 (0.67-0.76)	0.76 (0.70-0.81)	0.69 (0.63-0.74)
	Net4	0.76 (0.71-0.80)	0.72 (0.67-0.76)	0.74 (0.68-0.79)	0.68 (0.58-0.78)
	Net5	0.79 (0.73-0.84)	0.74 (0.69-0.79)	0.77 (0.74-0.81)	0.70 (0.67-0.72)
DREAM5	*InSilico*	0.84 (0.83-0.65)	0.85 (0.83-0.86)	0.86 (0.84-0.87)	0.86 (0.85-0.88)

Supplementary Table 3. **Comparison between the performance of SVM classifier and RFs classifier on graph-based features based on area under the ROC curve (AUC) and area under the Precision-Recall curve (AUPR).** The extent to which the choice of machine learning algorithms affects the performance of the model is assessed by applying SVM and RFs classifier. The numbers in parentheses refer to confidence intervals for AUC and AUPR values (see Methods). The comparison includes the five synthetic datasets from the DREAM4 challenge as well as the one synthetic data from the DREAM5 challenge.

**Data**		**Methods**			
		SVM on graph-based features		RFs on graph-based features	
		AUC	AUPR	AUC	AUPR
DREAM4	*Net1*	0.86 (0,80-0,91)	0.81 (0.76-0.86)	0.78 (0.74-0.82)	0.59 (0.57-0.61)
	Net2	0.79 (0.87-0.83)	0.78 (0.75-0.82)	0.76 (0.73-0.79)	0.54 (0.52-0.56)
	Net3	0.77 (0.75-0.80)	0.72 (0.67-0.76)	0.62 (0.59-0.65)	0.49 (0.48-0.50)
	Net4	0.76 (0.71-0.80)	0.72 (0.67-0.76)	0.62 (0.60-0.65)	0.52 (0.50-0.54)
	Net5	0.79 (0.73-0.84)	0.74 (0.69-0.79)	0.65 (0.62-0.68)	0.52 (0.50-0.54)
DREAM5	*InSilico*	0.84 (0.83-0.65)	0.85 (0.83-0.86)	0.70 (0.69-0.70)	0.53 (0.53-0.54)

Supplementary Table 4. **The size of training/testing data sets.** The size of training data sets used to train SVM are presented for five synthetic datasets from the DREAM4 challenge as well as the one synthetic and the two real-world datasets from the DREAM5 challenge. In all training sets 90% of the positive instances are randomly selected from the positive sets. To keep the balance between positive and negative instances, the same number of negative instances are also selected randomly (hence the “×2” in the respective table entries).

**Data**		Size of positive set	Size of training set	Size of testing set
DREAM4	*Net1*	176	158×2 = 316	18×2 = 36
	Net2	249	224×2 = 448	25×2 = 50
	Net3	195	175×2 = 350	20×2 = 40
	Net4	211	189×2 = 378	22×2 = 44
	Net5	193	173×2 = 346	20×2 = 40
DREAM5	*InSilico*	4012	3610×2 = 7220	402×2 = 804
	E. coli	2066	1859×2 = 3718	207×2 = 414
	S. cerevisiae	3940	3546×2 = 7092	394×2 = 788
